# Evaluation of changes in antimicrobial susceptibility in bacteria infecting children and their mothers in pediatric, neonatal-intensive care unit, and gynecology/obstetrics wards of a quaternary referral hospital during the COVID-19 pandemic

**DOI:** 10.3389/fmicb.2023.1096223

**Published:** 2023-02-20

**Authors:** Felipe Ramos Pinheiro, Rafaela Elvira Rozza-de-Menezes, Marina Camille Blum, Renata Freire Alves Pereira, Jaqueline Abel Rocha, Maria Cristina F. Guedes Pinto, Bruno A. Penna, Lee W. Riley, Fabio Aguiar-Alves

**Affiliations:** ^1^Molecular Epidemiology and Biotechnology Laboratory, School of Pharmacy, Fluminense Federal University, Niteroi, Brazil; ^2^Pathology Post Graduate Program, School of Medicine, Fluminense Federal University, Niteroi, Brazil; ^3^Division of Health and Social Behavior, School of Public Health, University of California, Berkeley, Berkeley, CA, United States; ^4^Applied Microbiology and Parasitology Post Graduate Programs, Fluminense Federal University, Niteroi, Brazil; ^5^Epidemiological Surveillance Unit, Antonio Pedro University Hospital, Fluminense Federal University, Niteroi, Brazil; ^6^Gram-Positive Cocci Laboratory, Biomedical Institute Fluminense Federal University, Niteroi, Brazil; ^7^Division of Infectious Diseases and Vaccinology, School of Public Health, University of California, Berkeley, Berkeley, CA, United States; ^8^Department of Pharmaceutical Sciences, Lloyd L. Gregory School of Pharmacy, Palm Beach Atlantic University, West Palm Beach, FL, United States

**Keywords:** antimicrobial resistance, COVID-19, bacterial infections, MDR, pediatrics

## Abstract

The World Health Organization released a statement warning of increased risk for the incidence of multidrug resistant microorganisms and the absence of new drugs to control such infections soon. Since the beginning of the COVID-19 pandemic, the prescription of antimicrobial agents has increased and may have accelerated the emergence of multidrug resistant (MDR) bacteria. This study aimed to evaluate maternal and pediatric infections within a hospital from January 2019 to December 2021. An observational retrospective cohort study was performed at a quaternary referral hospital in a metropolitan area of Niteroi city, Rio de Janeiro state, Brazil. A total of 196 patients’ medical records were analyzed. The data from 90 (45.9%) patients were collected before the SARS-CoV-2 pandemic, 29 (14.8%) from the 2020 pandemic period, and 77 (39.3%) from the 2021 pandemic period. A total of 256 microorganisms were identified during this period. Out of those, 101 (39.5%) were isolated in 2019, 51 (19.9%) in 2020, and 104 (40.6%) in 2021. Antimicrobial susceptibility tests were performed on 196 (76.6%) clinical isolates. The exact binomial test showed that the distribution of Gram-negative bacteria was predominant. The most common microorganism was *Escherichia coli* (23%; *n* = 45), followed by *Staphylococcus aureus* (17.9%, *n* = 35), *Klebsiella pneumoniae* (12.8%, *n* = 25), *Enterococcus faecalis* (7.7%, *n* = 15), *Staphylococcus epidermidis* (6.6%, *n* = 13) and *Pseudomonas aeruginosa* (5.6%, *n* = 11). *Staphylococcus aureus* was the predominant species among resistant bacteria. Among the antimicrobial agents tested, the following were resistant, presented on a descending scale: penicillin (72.7%, *p* = 0.001, Binomial test), oxacillin (68.3%, *p* = 0.006, Binomial test), ampicillin (64.3%, *p* = 0.003, Binomial test), and ampicillin/sulbactam (54.9%, *p* = 0.57, Binomial test). Infections with *S. aureus* were 3.1 times greater in pediatrics and maternal units than in other hospital wards. Despite the global reduction in the incidence of MRSA, we observed an increase in MDR *S. aureus* in this study. No changes were observed in the frequency of resistance profiles of the clinical isolates after the establishment of the global SARS-CoV-2 pandemic. More comprehensive studies are needed to understand the impact of the global SARS-CoV-2 pandemic on the resistance levels of bacteria associated with neonate and pediatric patients.

## 1. Introduction

In the past 3 years, the world has been facing a pandemic caused by the severe acute respiratory syndrome coronavirus 2 (SARS-CoV-2). The first confirmed SARS-CoV-2 infection in Latin America occurred in the state of São Paulo in February 2020 (Rodriguez-Morales et al., 2020). The coronavirus disease 2019 (COVID-19) culminated in a global public health emergency, posing significant challenges to healthcare professionals and great loss of life. This situation has led to countless hospitalizations in intensive care units (ICUs), and increased use of ventilatory support (Guan et al., 2020; Saeed et al., 2021). Viral infections are commonly associated with bacterial co-infection, leading to an increase in morbidity and mortality. Such co-infections are well recognized with influenza and COVID-19, which increased the non-discriminatory use of antimicrobial agents during the pandemic (Lansbury et al., 2020; Saeed et al., 2021).

The World Health Organization (WHO) classifies antimicrobial-resistant (AMR) microorganisms as a threat to human health (WHO 2014). According to WHO: *Enterococcus faecium*, *Staphylococcus aureus*, *Klebsiella pneumoniae*, *Acinetobacter baumannii*, *Pseudomonas aeruginosa*, *Enterobacter* spp., and *Escherichia coli*, are cumulatively known as ESKAPEEc group. These microorganisms represent a critical role in nosocomial infections due to their virulence and acquisition of resistance to various antimicrobial agents, including those used as the last resource in ICUs, which result in increases in cost and length of stay of the patients in the hospital environment. In particular, increased antibiotic resistance contributes to a growing shortage of drugs capable of treating these pathogens (Rice 2008; WHO 2017; De Angelis et al., 2018; Gaspari et al., 2021).

The rising incidence of antimicrobial-resistant infections represents a global public health concern (Romandini et al., 2021). The World Health Organization estimates that multidrug resistant microorganisms (MDR) cause 700,000 deaths annually. Newborns represent approximately 30% (210,000) of these deaths (WHO 2016). These MDR bacteria are frequently associated with more severe infections, long-term hospitalizations, and higher treatment costs than those not presenting opportunistic resistance. As a result, MDR bacteria have proven an increased mortality rate of up to 40% (Naylor et al., 2018; Johnston et al., 2019; Romandini et al., 2021).

Since a significant increase in the use of antimicrobial agents along with other alternative medications has been reported during the pandemic, concerns related to the indiscriminate use of such medications are also rising (Lansbury et al., 2020; Vestesson et al., 2022). Specifically, increased rates of AMR may result from empirical treatment with antimicrobial agents without isolating or testing for etiologic microorganisms (Rawson et al., 2020).

Additionally, recent reports show a significant increase in nosocomial infections in neonates associated with MDR bacteria (Mammina et al., 2007; Patel and Saiman 2010; Silva et al., 2021), increasing newborn’s risk of developing complications related to infection by pathogenic AMR microorganisms. Given that the threat of MDR infections for newborns may already be rising, exacerbation of this trend due to antimicrobial use during the COVID-19 pandemic is of great concern (Mammina et al., 2007; Rawson et al., 2020).

There is a lack of Brazilian studies on the resistance profiles of bacterial isolates to antibiotics in pediatric and maternal units. This study aims to retrospectively investigate changes in antimicrobial resistance among bacterial isolates collected before and during the COVID-19 pandemic. Specifically, this study will focus on pediatric, gynecology/obstetrics, and neonatal ICU patients at a quaternary referral university hospital in the metropolitan region of Niteroi city, in Rio de Janeiro.

## 2. Materials and methods

### 2.1. Ethical approval statement

This study was submitted and approved by the Research Ethics Committee of the Faculty of Medicine of the Universidade Federal Fluminense (CEP – FM/UFF) and by CONEP, being registered under the number CAAE: 26823614.2.0000.5243. To maintain confidentiality, the data used were anonymized.

### 2.2. Study design and data analysis

We performed a single-center retrospective observational study on pediatric, neonatal-ICU, and gynecology/obstetrics wards. Data from January 1, 2019, to December 31, 2021, were provided by Hospital Infection Control Committees’ (HICC) reports at the Antonio Pedro University Hospital (HUAP), which is a quaternary referral hospital according to the Brazilian public health system (SUS). HUAP is the main COVID-19 treatment center in the Rio de Janeiro metropolitan area II, covering seven cities and serving approximately two million people. All patients admitted to HUAP within the groups in the three-year period were evaluated, regardless of whether they had hospital-acquired infections.

To avoid bias regarding reducing admission to pediatric, neonatal-ICU, and gynecology/obstetrics wards, all elective surgeries and outpatient care were suspended, leaving the unit for the complete care of COVID-19 patients.

We divided data into three periods of the pandemic in Brazil: January 2019 to February 2020, March 2020 to December 2020, and January 2021 to December 2021.

### 2.3. Study population

All patients admitted to the gynecology/obstetrics, pediatrics and NICU registered by the HICC were included in this study.

Medical records data obtained included the profile of patients (women or neonate/pediatric patients); site of isolation (sterile or non-sterile); and bacteria species including Gram classification profile, resistance profile, and period of isolation. We identified all clinical isolates, including those from colonization and infection sites, however only strains retrieved from infection sites were subjected to an antimicrobial susceptibility test (AST) according to HICC protocols.

All women attending the gynecology and obstetrics clinics were included in the study. All pediatric patients (including neonates) admitted to HUAP in the period were also evaluated in the present study. Thus, the study analyzed 196 patients’ medical records, divided into 36 (18.4%) women from obstetrics, and 160 (81.6%) neonate or pediatric patients. The data from 90 (45.9%) patients were collected before the SARS-CoV-2 pandemic, 29 (14.8%) from the 2020 pandemic period, and 77 (39.3%) from the 2021 pandemic period.

### 2.4. Species identification and antimicrobial susceptibility testing

The antimicrobial resistance profiles were provided by Phoenix BD automated system (Becton Dickinson Franklin Lakes, NJ, EUA); according to manufacturing protocols, each panel was standardized for Gram-positive and Gram-negative AST profiles comprehending the list below:

*Aminoglycoside*: Amikacin (AMK), Gentamicin (GEN), Synergism Gentamicin (SGEN), Synergism Streptomycin (SSTP), Tobramycin (TOB); *Cephalosporins*: Cefepime (FEP), Cefoxitin (FOX), Ceftaroline (CPT), Ceftazidime (CAZ), Ceftazidime + Avibactam (CZA), Ceftriaxone (CRO), Cefuroxime (CXM), Cefazolin (CZ); *Quinolones*: Ciprofloxacin (CIP), Norfloxacin (NX), Levofloxacin (LVX); *Penicillin*: Amoxicillin/Clavulanic acid (AMC), Ampicillin (AMP), Ampicillin/Sulbactam (SAM), Oxacillin (OXA), Penicillin (PEN), Piperacillin/Tazobactam (TZP); *Carbapenems*: Ertapenem (ETP), Imipenem (IPM), Meropenem (MEM); *Glycopeptides*: Teicoplanin (TEC), Vancomycin (VAN): *Macrolide*: Erythromycin (ERY), Rifampicin (RIP): *Lincosamides*: Clindamycin (CLI); *Oxazolidinone*: Linezolid (LZD); *Tetracycline*: Tetracycline (TET), Minocycline (MIN); *Sulfonamides*: Sulfamethoxazole/Trimethoprim (STX); *Nitroimidazoles*: Nitrofurantoin (NIT); *Amphenicol*: Chloramphenicol (C); *Phosphonate*: Fosfomycin (FOS); *Glycylcyclines*: Tigecycline (TGC); *Polypeptide*: Colistin (CL); *Lipopeptides*: Daptomycin (DAP).

The resistance profile was classified as resistant (R), and susceptible (S). Any isolate with resistance to three or more classes of antimicrobial agents was classified as multidrug-resistant (MDR) according to the definition proposed by Magiorakos et al. (2012). Some of the clinical isolates were retrieved at the moment of hospitalization for epidemiological active surveillance and infection control. A total of 256 isolates were included in the study and 196 had the antimicrobial susceptibility test performed (Table 1).

**Table 1:** Clinical isolates identified at Antonio Pedro University Hospital during the study period.

Microorganism	Pre-pandemic			2020			2021		
	Total	AST	MDR	Total	AST	MDR	Total	AST	MDR
*Acinetobacter baumannii*	1	1	1	-	-	-	1	1	0
*Bacillus coagulans*	-	-	-	1	0	0	-	-	-
*Bacillus megaterium*	-	-	-	1	0	0	-	-	-
*Burkholderia cepacia* complex	-	-	-	-	-	-	1	0	0
*Burkholderia gladioli*	-	-	-	-	-	-	1	0	0
*Citrobacter freundii*	-	-	-	-	-	-	1	1	0
*Citrobacter koseri*	2	2	0	1	1	0	1	1	0
*Enterobacter aerogenes*	4	4	0	1	1	0	-	-	-
*Enterobacter cloacae*	3	3	0	-	-	-	2	2	0
*Enterobacter cloacae* complex	1	1	0	1	0	0	2	2	0
*Enterococcus faecalis*	5	5	3	6	4	1	6	6	0
*Escherichia coli*	19	17	5	8	7	1	25	21	7
*Haemophilus* sp.	2	1	0	-	-	-	1	1	0
*Kingella denitrificans*	-	-	-	-	-	-	1	0	0
*Klebsiella aerogenes*	-	-	-	2	2	0	-	-	-
*Klebsiella oxytoca*	1	1	0	-	-	-	2	1	0
*Klebsiella ozaenae*	-	-	-	-	-	-	1	1	1
*Klebsiella pneumoniae*	8	7	3	5	5	2	19	13	0
*Listeria monocytogenes*	-	-	-	1	1	0	-	-	-
*Paenibacillus* sp	-	-	-	-	-	-	1	0	0
*Pasteurella multocida*	-	-	-	-	-	-	1	0	0
*Proteus mirabilis*	1	1	0	1	1	0	2	2	0
*Providencia rettgeri*	-	-	-	-	-	-	1	1	0
*Pseudomonas aeruginosa*	7	5	2	5	2	0	5	4	2
*Salmonella* group	-	-	-	1	1	0	-	-	-
*Serratia marcescens*	1	1	0	-	-	-	1	1	0
*Sphingomonas paucimobilis*	-	-	-	-	-	-	1	0	0
*Staphylococcus aureus*	18	16	1	7	7	1	12	12	3
*Staphylococcus capitis*	2	1	0	1	0	0	1	1	1
*Staphylococcus caprae*	1	0	0	-	-	-	-	-	-
*Staphylococcus epidermidis*	11	9	4	3	0	0	8	4	3
*Staphylococcus haemolyticus*	4	2	1	2	1	0	1	1	1
*Staphylococcus hominis*	3	2	1	-	-	-	1	0	0
*Staphylococcus kloosii*	-	-	-	1	0	0	-	-	-
*Staphylococcus pasteuri*	1	1	0	-	-	-	-	-	-
*Staphylococcus warneri*	-	-	-	-	-	-	2	1	1
*Staphylococcus saprophyticus*	2	2	0	-	-	-	-	-	-
*Staphylococcus schleiferi*	1	0	0	-	-	-	-	-	-
*Stenotrophomonas maltophilia*	-	-	-	-	-	-	1	0	0
*Streptoccoccus agalactiae*	1	0	0	2	2	0	-	-	-
*Streptococcus gallolyticus* ssp *pasteurianus*	-	-	-	-	-	-	1	0	0
*Streptococcus oralis*	-	-	-	-	-	-	1	0	0
*Streptococcus pneumoniae*	1	1	0	-	-	-	-	-	-
*Streptococcus pyogenes*	-	-	-	1	1	0	-	-	-
*Streptococcus viridans*	1	0	0	-	-	-	-	-	-
Total	101	83	21	51	36	5	104	77	19

AST antimicrobial susceptibility test; MDR, Bacteria multidrug resistance; Total, represents a total number of clinical isolates identified, regardless of whether being tested or not.

Data for new COVID-19 cases for each month were obtained from the Brazilian Ministry of Health (MS) and the State Health Department of Rio de Janeiro, compiled by Cota (2020).

The prevalence of bacteria species in pediatric, neonatal-ICU, and gynecology/obstetrics wards during the pandemic period was evaluated. In order to compare these three wards with other hospital wards, a total of 2,551 bacteria isolates were recovered from the HICC-HUAP.

### 2.5. Statistical analysis

The Statistical Package for the Social Sciences Program (IBM^®^ SPSS^®^ Statistics, version 20.0, United States) and Jamovi Statistical Software [The Jamovi Project (2021), version 1.6.23.0, retrieved from https://www.jamovi.org] were used for statistical analysis considering the 0.05 level of statistical significance. The data processing, data cleaning, and preparation of tables were performed on R studio *Version 1.4.1717* (R 4.1.0). Graphs were prepared on GraphPad Prism 8.

Shapiro–Wilk normality test was performed to evaluate the normality of variables. The percentage of antimicrobial agents and classes of antibiotics with resistance profile; per isolate; and frequencies of Susceptibility, AMR, and MDR were calculated for the analysis into three time periods. A dichotomous outcome was defined for the three more common bacteria found during the pandemic to estimate the percentage of microorganisms. Thus, for example, isolates that showed *Staphylococcus aureus* contamination would have the rest of the isolates classified as “without this bacterium” for the analysis 2 × 2 to maximize the power to detect independent associations with the prevalence of bacteria species.

Also, categorical variables in the three time periods described above were compared using Pearson’s Chi-square test or Fisher’s exact test, with Monte Carlo simulation to estimate the value of *p*s.

The binomial test was used for categorical intragroup comparisons. Kruskal-Wallis and Mann–Whitney tests were selected for independent analyzes of numerical variables. Ordinal and Multivariate linear regression models using ‘enter’ entry procedures were used to assess the independent effects of variables on the number of antimicrobial agents per isolate and resistance profile (frequencies of S, AMR, and MDR). For the final model, the variables were considered significant if they had a value of *p* ≤ 0.05 after adjustment.

## 3. Results

Two hundred fifty-six microorganisms were isolated from the pediatric, neonatal-ICU, and gynecology/obstetrics wards from 2019 to 2021. From these, 101 (39.5%) were isolated in 2019, 51 (19.9%) in 2020, and 104 (40.6%) in 2021. The antimicrobial susceptibility tests were performed on a total of 196 (76.6%) clinical isolates (the other 60 isolates were obtained from the epidemiological inquiry). Ninety (45.9%) isolates were collected before the SARS-CoV-2 pandemic, 29 (14.8%) from the 2020 pandemic period, and 77 (39.3%) from the 2021 pandemic period (Table 1). The medical records were composed of 36 (18.4%) women and 160 (81.6%) neonates/pediatric patients, of which 19 (9.6%) women and 87 (44.4%) neonates/pediatric patients were sampled during the pandemic period.

A total of 30 distinct species were identified in this study. The most common species was *Escherichia coli* (23%; *n* = 45), followed by: *Staphylococcus aureus* (17.9%, *n* = 35), *Klebsiella pneumoniae* (12.8%, *n* = 25), *Enterococcus faecalis* (7.7%, *n* = 15), *Staphylococcus epidermidis* (6.6%, *n* = 13) and *Pseudomonas aeruginosa* (5.6%, *n* = 11). During the period before the pandemic, the main species isolated was *Escherichia coli* (20%), followed by *Staphylococcus aureus* (17.8%). A similar feature has been seen in 2020 with a higher frequency for *Staphylococcus aureus* (24.1%), followed by *E. coli* (20.7%). In 2021, the prevalent species was *E. coli* (27.3%), and *K. pneumoniae* was the second most common species with 16.9%, followed by *S. aureus* (15.6%). There was a notable increase in this Enterobacteria species during the global SARS-CoV-2 pandemic, reaching a frequency 2-fold higher than in 2019 (16.9% versus 8.9%). Apart from the increase in *K. pneumoniae*’s incidence, none of the most common species of bacteria had a significant frequency rise during the pandemic (*p* = 0.669, Pearson chi-square test).

There was a predominance of Gram-negative bacteria (59.2%, *n* = 116) using the data from 196 isolates (*p* = 0.01, Exact binomial test). The clinical isolates were classified as Gram-negative (GN) bacteria in 48 (53.4%) isolates before the pandemic period, 16 (55.2%) in the 2020 pandemic period, and 52 (67.5%) in the 2021 pandemic period. There were no significant changes in GN bacteria when comparing the three periods (*p* = 0.20, Pearson chi-square test). On the other hand, when analyzing the strains separately by period, only the 2021 pandemic year (*n* = 77) revealed a significant proportion of GN microorganisms (*p* = 0.003 versus *p* = 0.59/*n* = 90 and *p* = 0.71/*n* = 29 to before and 2020 pandemic periods, respectively).

Neonates and pediatric patients showed high *Staphylococcus aureus* infections from the sterile fluid collection during the pandemic. *S. aureus* was more prevalent in neonates/pediatric patients than women (97.1% versus 2.8%, *p* = 0.014, OR = 9.444, IC 95% 1.2–71.4, Pearson chi-square test). However, neonates/pediatric patients had a lower risk for *E. coli* infection (*p* < 0.0001, OR = 0.203, IC 95% 0.09–0.44, Pearson chi-square test). Women exhibited significant GN bacteria contamination, regardless of the period (*n* = 196, 80.6% of women versus 54.4% of neonates/pediatric patients, *p* = 0.004, OR = 3.476, IC 95% 1.439–8.398, Pearson chi-square test).

Sterile sites were considered a protective factor for *E. coli* infection in approximately 84% of isolates (91.1% in non-sterile versus *37.7*% in a sterile area with this bacteria diagnosis; *p* < 0.0001, OR = 0.078, IC 95% 0.01–0.61, Pearson chi-square test). The non-sterile sites were responsible for significant variability in GN bacteria phenotypes, showing a 5-fold increase in frequencies compared to Gram-positive (GP) bacteria (*p* < 0.0001, OR = 5.046, IC 95% 2.631–9.679, Pearson chi-square test).

**GRAPH 1:**
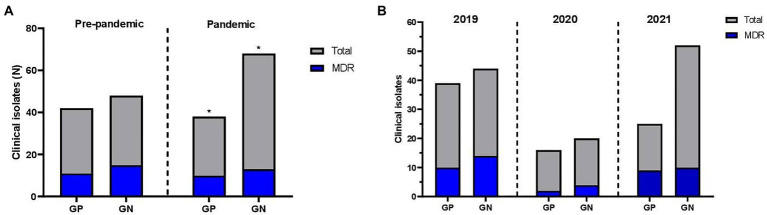
Frequencies of Multidrug Resistance of clinical isolates in pediatric and maternal units of Antonio Pedro University Hospital according to periods of study **(A)** Before and during the pandemic and **(B)** by year.

**GRAPH 2:**
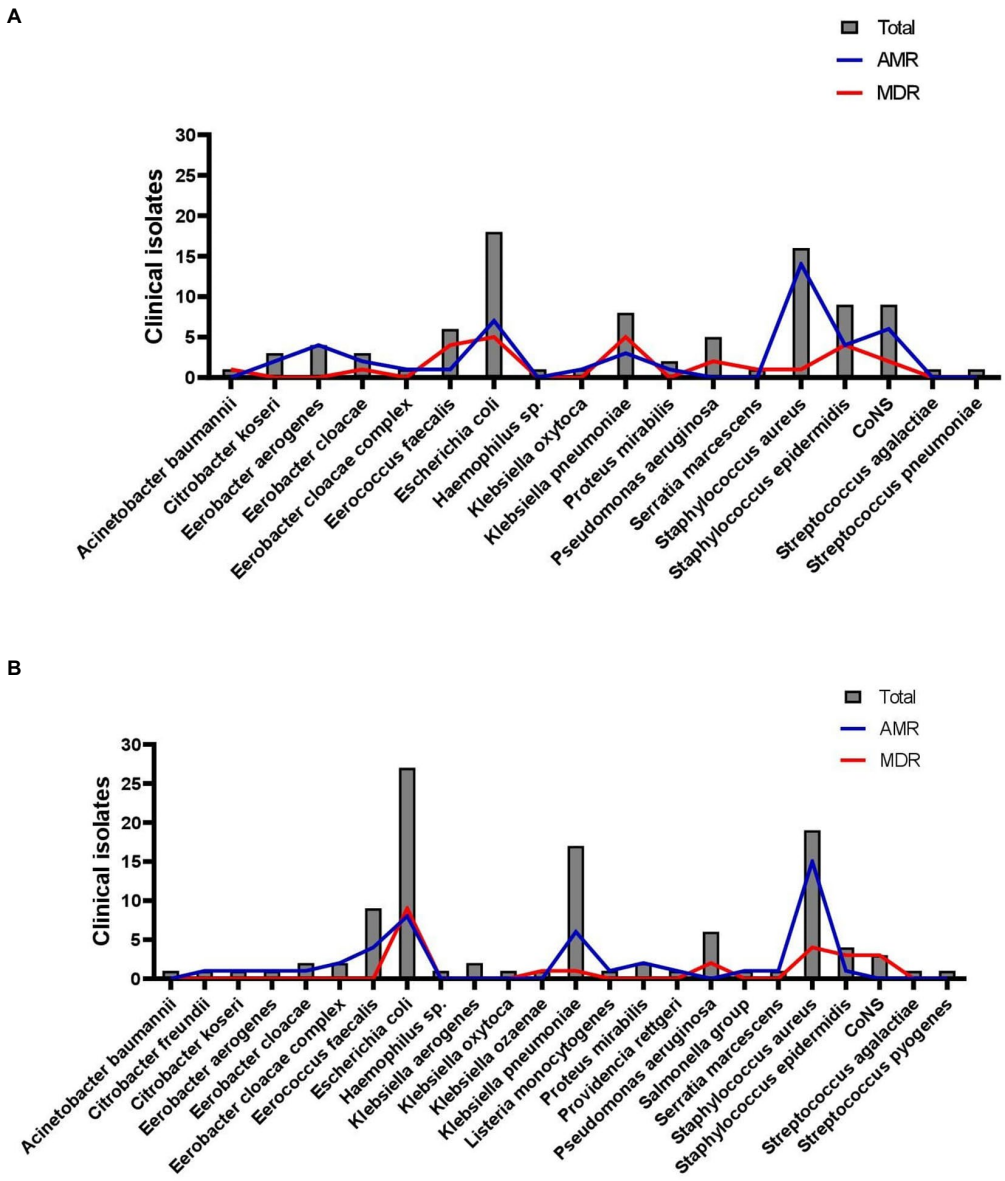
Clinical isolates resistant profile identified among pediatric and maternal units of Antonio Pedro University Hospital. **(A)** Pre-pandemic period; **(B)** COVID-19 pandemic period.

Graph 1A reveals the frequency of MDR bacteria before the COVID-19 pandemic and during the first pandemic year. Graph 1B shows the frequency of MDR bacteria by year. The antimicrobial agents had a decrease in the frequency of resistance during the pandemic. A total of 39 antibiotics from 18 classes were included in the present study. The results of the susceptibility tests are shown in Table 1. Approximately 72% (*n* = 141) of the isolates were resistant to one of the antimicrobial agents tested, of which 46.9% (*n* = 92) showed AMR outcomes, and 25% (*n* = 49) were classified as MDR. Finally, susceptibility to all antimicrobial agents tested was seen in 28.1% (*n* = 55) of the isolates (Graphs 2A,B).

Considering the MDR isolates, 19 (9.7%) were resistant to three classes of antibiotics, 18 (9.2%) to four, 10 (5.1%) to five, and two (1%) to six. Antimicrobial agents of the penicillin class presented the highest number of resistant isolates (*n* = 124; 63.3%). There were no statistically significant differences in the frequency of S, AMR, and MDR cases among isolates collected from these three periods of this study (frequency of MDR in average period, 2020 and 2021 pandemic periods: 13.3, 2.0, and 9.7%), as shown in Graphs 1A,B. In opposition, there was a little increase in susceptibility (S) in the 2021 pandemic period (frequency of “S” in the standard 2020 and 2021 pandemic periods: 9.2, 6.1, and 12.8%).

There was a variation in the resistance to antimicrobial agents ranging from 2.6% (*n* = 1) to 33.3% (*n* = 13) per isolate (6.7 ± 6.6%). Regarding classes of antimicrobials, a minimum of 5.5% (*n* = 1) and a maximum of 33.3% (*n* = 6) of classes of antimicrobial agents presenting resistance per isolate (9.0 ± 8.5%). The number of isolates presenting antimicrobial resistance decreased during the pandemic among the three study periods (*p* = 0.02, Kruskal-Wallis test), however, the classes did not significantly reduce when comparing the periods (*p* = 0.06; Kruskal-Wallis test).

Regarding the most common species of this study (*Escherichia coli*, 23%; *Staphylococcus aureus*, 17.9%; *Klebsiella pneumoniae*, 12.8%; *Enterococcus faecalis*, 7.7%; *Staphylococcus epidermidis*, 6.6%; *Pseudomonas aeruginosa*, 5.6%), *Staphylococcus aureus* species presented the higher resistance rates during 2019 and pandemic (*p* = 0.002 and *p* < 0.0001, respectively, Fisher’s exact test), and there was no *S. aureus* susceptible during the pandemic. *S. aureus* species (*n* = 35) was predominant among resistant bacteria, accounting for 29 isolates (82.9% of the *S. aureus* cases, *p* > 0.0001, Fisher’s exact test). Of these 29 isolates, five (14.2%) were MDR. *S. aureus* had a high resistance rate to the Penicillin class (97%, *p* < 0.0001), which the 35 isolates were tested for penicillin drug with 94% of resistance (*p* < 0.0001, Binomial test), and nine isolates of *S. aureus* were tested for ampicillin drug with 100% of resistance rates (*p* = 0.004, Binomial test). Cephalosporin (*S. aureus* tested: 100% of isolates with 97% of S), carbapenem (*S. aureus* tested: 100% of isolates with 100% of S), aminoglycosides (*S. aureus* tested: 100% of isolates with 89% of S), quinolones (*S. aureus* tested: 100% of isolates with 97% of S), glycylcycline (*S. aureus* tested: 72% of isolates with 100% of S), sulfonamide (*S. aureus* tested: 100% of isolates with 97% of S), glycopeptide (*S. aureus* tested: 100% of isolates with 100% of S), macrolides (*S. aureus* tested: 100% of isolates with 83% of S), lipopeptides (*S. aureus* tested: 100% of isolates with 100% of S), oxazolidone (*S. aureus* tested: 100% of isolates with 97% of S), lincosamide (*S. aureus* tested: 52% of isolates with 78% of S), tetracycline (*S. aureus* tested: 48.5% of isolates with 94% of S) and nitroimidazoles (*S. aureus* tested: 5.8% of isolates with 100% of S) were tested for *S. aureus* without detachable resistance rates.

The *E. coli* and *K. pneumoniae* isolates did not demonstrate significant AMR or MDR profiles (*p* = 0.114 and *p* = 0.317, respectively, Pearson chi-square test). Out of 80 Gram-positive isolates, 57.5% (*n* = 46), of which 36.25% were *S. aureus*, presented an AMR profile (*p* = 0.006, Pearson chi-square test). This frequency rose significantly during the SARS-CoV-2 pandemic (value of *p* in regular and pandemic periods: 0.287 and 0.028, Pearson chi-square test).

Concerning the susceptibility to antibiotics in all isolates tested it was observed a higher resistance frequency than susceptibility on a descending scale: penicillin (72.7%, *p* = 0.001, Binomial test), oxacillin (68.3%, *p* = 0.006, Binomial test), ampicillin (64.3%, *p* = 0.003, Binomial test), and ampicillin/sulbactam (54.9%, *p* = 0.57, Binomial test). The other 34 drugs revealed a higher susceptibility frequency.

Neonates or pediatric patients covered 93.9% of the MDR cases (*p* = 0.01; Pearson chi-square test). After separating the data per period, the highest MDR rate has been found among patients from the pandemic period (*p* = 0.006, *n* = 106, versus *p* = 0.56 before the pandemic, *n* = 90, Pearson chi-square test). The possible explanation for this observation comes from the knowledge that 100% (*n* = 23) of the MDR microorganisms were collected from neonates or pediatric patients. Isolates collected from women showed a predominance (63.2%, *n* = 12) in terms of susceptibility during the pandemic as well.

The 2,251 exams collected by HICC-HUAP in 2021 revealed a 3-fold risk of infections by *S. aureus* acquired in the pediatric, neonatal-ICU, and gynecology/obstetrics wards (*S. aureus*: 6.2% versus 17.9% for all bacteria species, OR = 3.158, IC 95% 1.8–5.3, *p* < 0.0001, Pearson chi-square test).

The results of the ordinal and multivariate linear regression analyzes are shown in Table 2. Seven variables were entered into the models. Neonates/pediatric patients (*p* = 0.04) and the pandemic period (*p* = 0.008) showed a significant probability of presenting resistant isolates. The model is statistically significant (Χ^2^ = 18.402, df = 7, *p* = 0.01), and the corresponding value for *R*^2^ was 0.102. The variables associated with the number of antimicrobial agents and the resistance per isolate after adjustments were neonatal/pediatric patients (*p* = 0.001) and the pandemic period (*p* = 0.01). The model was also significant (*F*(7, 188) = 2.757; *p* = 0.009), and the *R*^2^ value associated with it was also 0.102.

**Table 2:** Results of the multiple ordinal regression and multivariate linear regression analysis about antimicrobial resistant and multi-resistant drugs.

Dependent variable: resistance profile (ordinal) *R*^2^ (Nagelkerke)=0.102; *R*²=18.402;df=7; *p*=0.01 independent variables	Estimate (*β*₀)	Standard error	*p*
Period of the study (before or during the pandemia)	−0.572	0.278	0.04
*Escherichia coli* species	0.625	0.411	0.12
*Klebsiella pneumoniae* species	0.023	0.477	0.96
*Staphylococcus aureus* species	0.020	0.435	0.96
Gram profile of bacterias	0.472	0.423	0.26
Patient profile	1.039	0.394	0.008
Collected fluid site	0.247	0.333	0.45
Dependent variable: number of resistant antimicrobial agents per isolate (linear) *R*^2^=0.102; *F*(7,188)=3.064; *p*=0.004 independent variables	Exp (*β*)	95% I.C.	*p*-value
Period of the study (before or during the pandemia)	−2.438	−4.3 to −0.59	0.01
*Escherichia coli* species	1.082	−1.6–3.8	0.43
*Klebsiella pneumoniae* species	3.593	1.2–11.6	0.032
*Staphylococcus aureus* species	0.998	0.98–1.0	0.209
Gram profile of bacterias	−0.603	−3.4−2.2	0.67
Patient profile	−4.243	−6.8 to −1.6	0.001
Collected fluid site	−1.016	−3.2–1.2	0.37

## 4. Discussion

Since the beginning of the SARS-CoV-2 pandemic, several studies regarding hospital-associated infections (HAIs) in the context of COVID-19 have been published (Karaba et al., 2021; O’Toole 2021; Salem et al., 2021; Baker et al., 2022). Our group analyzed 196 clinical isolates from patients hospitalized at the HUAP from the obstetric, gynecological, pediatric, and neonatal ICU wards.

A comprehensive systematic review and meta-analysis study identifies an incidence of 569 (29%) antimicrobial-resistant organisms. Among MDR bacteria, MRSA, carbapenem-resistant *A. baumannii, and Klebsiella pneumoniae* were commonly reported (Kariyawasam et al., 2022).

*Escherichia coli* has emerged as one of the leading causes of maternal sepsis and bacteremia during pregnancy (Robbins and Bakardjiev 2012; Surgers et al., 2014; Knowles et al., 2015). The WHO Global Maternal Sepsis Study (GLOSS) points out that urinary tract infection is the primary source of sepsis during pregnancy (Bonet et al., 2020). Surgers et al. (2014), suggested that *E. coli*, a significant commensal of the urinary tract, is responsible for approximately 27% of fetal mortality. Besides being the prevalent microorganism infecting subjects within the 3 years of this study, *E. coli* isolates showed an increase in MDR, and it was observed an increase in ampicillin resistance during the studied period. There is a lack of Brazilian studies, mainly in pediatric and neonatal wards, to be used as references to support the present study (Silva et al., 2021).

*Staphylococcus aureus* presented a significant incidence of antibiotic-resistant isolates (86.4%). However, no changes in resistance profile were observed in this study. The risk for infection associated with *S. aureus* in maternal and pediatric units was three times greater than in other hospital wards. On the other hand, Jernigan et al. (2020), observed a 20.5% decrease in the incidence of MRSA isolates both in a hospital-onset and in communities.

According to Mędrzycka-Dąbrowska et al. (2021), carbapenem-resistant *Klebsiella pneumoniae* infections associated with COVID-19 are a significant concern in hospital facilities. Rational antibiotic therapy should be mandatory to prevent the increase of bacterial resistance and continuous control and surveillance of hospital infections caused by MDR organisms. We also observed an increase of *K. pneumoniae* during the SARS-CoV-2 pandemic period being more common in women because they were more commonly infected by GN microorganisms. Also, the increased incidence of MDR microorganisms was not related to the increase in Covid-19 cases in Rio de Janeiro.

In conclusion, our study shows a *S. aureus-*acquired infection in the pediatric and neonatal-ICU wards during the pandemic period. Neonates and pediatric patients were infected by multidrug resistant microorganisms during the pandemic, regardless of the diminished resistance profiles of the clinical isolates at HUAP, after establishing the global SARS-CoV-2 pandemic. More comprehensive studies are needed to understand the impact of the global SARS-CoV-2 pandemic on the high levels of multidrug resistance bacteria associated with neonates and pediatric patients and to investigate their relationship with acquired infection by *S. aureus* and other microorganisms associated with the hospital environment.

This study has limitations, like the reduced number of patients enrolled in 2020 due to the suspension of elective surgeries at this facility. In addition, we cannot access data regarding patients’ length of stay, their outcomes, or comorbidities.

## Data availability statement

The raw data supporting the conclusions of this article will be made available by the authors, without undue reservation.

## Ethics statement

The studies involving human participants were reviewed and approved by this study was approved by the Research Ethics Committee of the Faculty of Medicine of the Universidade Federal Fluminense (CEP – FM/UFF) and by CONEP, being registered under the number CAAE: 26823614.2.0000.5243. Written informed consent to participate in this study was provided by the participants’ legal guardian/next of kin.

## Author contributions

FP wrote the original draft, performed data analysis, and edited the manuscript. RR-M and MB performed data analysis and reviewed the manuscript. RP reviewed and edited the manuscript. JR and MP: data curation. BP: investigation and resources. LR final review of the manuscript. FA-A: conception, review of the manuscript, and investigation. All authors contributed to the article and approved the submitted version.

## Funding

PDPA-Fluminense Federal University and Niteroi City, FOPESQ-Fluminense Federal University (UFF).

## Conflict of interest

The authors declare that the research was conducted in the absence of any commercial or financial relationships that could be construed as a potential conflict of interest.

## Publisher’s note

All claims expressed in this article are solely those of the authors and do not necessarily represent those of their affiliated organizations, or those of the publisher, the editors and the reviewers. Any product that may be evaluated in this article, or claim that may be made by its manufacturer, is not guaranteed or endorsed by the publisher.
